# Micropuncture closure following high density microarray patch application in healthy subjects

**DOI:** 10.1111/srt.13131

**Published:** 2022-01-22

**Authors:** Joakim Henricson, David A. Muller, S. Ben Baker, Fredrik Iredahl, Totte Togö, Chris D. Anderson

**Affiliations:** ^1^ Department of Biomedical and Clinical Sciences Faculty of Health Sciences Linköping University Linköping Sweden; ^2^ Department of Emergency Medicine Local Health Care Services in Central Östergötland Linkoping Sweden; ^3^ School of Chemistry and Molecular Biosciences The University of Queensland St Lucia Queensland Australia; ^4^ Vaxxas Pty Ltd Translational Research Institute Woolloongabba Queensland Australia; ^5^ Department of Primary health care Region Östergötland Linköping Sweden; ^6^ Department of Medical and Health Sciences Faculty of Health Sciences Linköping University Linköping Sweden; ^7^ Allergy Centre Region Östergötland Linkoping Sweden; ^8^ Division of Cell Biology Faculty of Health Sciences Linköping University Linkoping Sweden

**Keywords:** evaporimetry, microneedles, polarisation reflectance spectroscopy, skin barrier integrity, skin reactivity, vaccination

## Abstract

**Background:**

The high‐density microarray patch (HD‐MAP) promises to be a robust vaccination platform with clear advantages for future global societal demands for health care management. The method of action has its base not only in efficient delivery of vaccine but also in the reliable induction of a local innate physical inflammatory response to adjuvant the vaccination process. The application process needs to induce levels of reactivity, which are acceptable to the vaccine, and from which the skin promptly recovers.

**Materials and methods:**

1 × 1 cm HD‐MAP patches containing 5000, 250‐μm long microprojections were applied to the skin in 12 healthy volunteers. The return of skin barrier function was assessed by transepidermal water loss (TEWL) and reaction to topical histamine challenge.

**Results:**

Skin barrier recovery by 48 h was confirmed for all HD‐MAP sites by recovered resistance to the effects of topical histamine application.

**Conclusions:**

Our previous observation, that the barrier disruption indicator TEWL returns to normal by 48 h, is supported by this paper's demonstration of return of skin resistance to topical histamine challenge in twelve healthy subjects.

## 1 INTRODUCTION

The world is currently experiencing an unprecedented severe acute respiratory syndrome corona virus 2 (SARS‐CoV‐2) pandemic causing COVID‐19 disease. As a result, several vaccine manufacturers have produced, tested and been granted emergency use of vaccines at an accelerated pace to fight SARS‐CoV‐2. The huge global demand for a vaccine to fight this newly emerged pathogen has identified several weak links in the global vaccine supply network. The need to keep messenger RNA vaccines very cold^1^ and the global shortage of glass vaccine vials^2^ pose real challenges to getting the vaccines into arms, particularly in resource poor settings. High‐density microarray patches (HD‐MAP) provide an alternative platform for vaccine delivery to the traditional needle and syringe for use in mass vaccination settings relevant for pandemic health care management.

**FIGURE 1:**
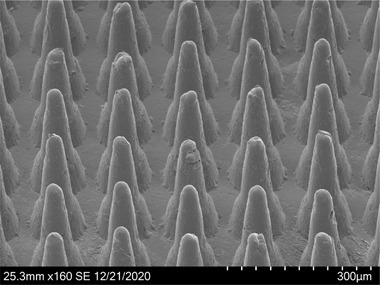
Scanning electron microscope image of the microneedles used in the present study

The HD‐MAP, constituted by a non‐dissolvable liquid crystal polymer, is a small 1 × 1 cm patch containing 5000 microprojections with a length of 250 μm on to which vaccine is dried (Figure 1). Each microprojection is conically shaped with a diameter of 120 μm at base and 25 μm at the tip and has an approximated penetration depth of 100 μm. When an HD‐MAP is applied to the skin, vaccine is deposited into the skin. This approach has been successfully demonstrated in pre‐clinical studies in mice and rats for a wide range of vaccine types including split virion,^3^ subunit vaccine,^4^ protein conjugate,^5^ inactivated virus^6^ and DNA.^7^ More recently the potential of the HD‐MAP has been demonstrated in phase 1 studies with influenza in humans^8^, ^9^ and a dengue fever vaccine candidate in mice.^10^ The HD‐MAP has several key advantages for use in mass vaccination settings such as: vaccines dried onto the HD‐MAP are thermostable^9^ not requiring refrigeration; they are easy to apply and do not require highly trained medical personal to administer the vaccine; the vaccine is delivered to the layer of the skin rich in antigen presenting cells resulting in a potentially faster and stronger immune response with a fractional dose when compared to needle‐based vaccine delivery.^11^

The HD‐MAP is applied to the skin using an applicator, which accelerates the HD‐MAP towards the skin where the microprojections puncture the stratum corneum and deposit the vaccine in the epidermis and upper dermis. The action of the microprojections penetrating the skin results in a regular array of micropunctures affecting the skin barrier. We have recently demonstrated that transepidermal water loss (TEWL) peaks immediately following HD‐MAP removal and then subsequently falls to pre‐application levels within 48 h following application, indicating that water vapour is no longer able to escape the skin.^12^ Increased TEWL is regarded as one of the most important parameters for demonstration of breached skin barrier integrity.^13^, ^14^ Decreasing TEWL values indicate return of barrier function towards normal.^15^ An alternative method to demonstrate the skin's return of barrier function is offered by observation of the effect of the application of the vasoactive agent histamine. When applied to normal skin, no reaction is observed. If the barrier is defective, itch, flare and wheal are experienced. If vaccination is to be carried out in a resource poor setting, full understanding of the details of the recovery period including the return of practical barrier function is of great interest in regard to application safety as well as appropriate care for the HD‐MAP application site following vaccination. In this study, we confirmed by TEWL the adequate HD‐MAP application bilaterally to the forearm and deltoid regions. Skin barrier recovery by 48 h was confirmed for both sites by recovered resistance to the effects of topical histamine application.

## 2 MATERIALS AND METHODS

### 2.1 Study design

Twelve healthy participants without known skin disease were recruited to the study (six females, six males, 18–65 years of age). Written informed consent was obtained from each subject participating. They rested for 30 min prior to measurements to ensure that they had reached equilibrium with the testing room. Participants received a total of four HD‐MAPs applied to the left and right volar forearm and upper arm deltoid regions, applied as described by Muller et al.^12^ HD‐MAP application sites were free from hair, tattoos, scars and visible veins. Prior to HD‐MAP application, baseline TEWL levels using a Tewameter‐300 probe (Courage ± Khazaka electronic GmbH, Köln, Germany) were established at each application site. After TEWL measurement, the skin application sites were cleaned with an alcohol swab then compressed with a pre‐calibrated skin conditioning ring (Vaxxas Pty Ltd, Brisbane, Australia) to apply a force of 30 N. A pre‐loaded applicator device (Vaxxas Pty Ltd, Brisbane, Australia) was then docked into skin conditioning ring. The applicator was triggered to apply the HD‐MAP at a speed of 20 m/s. Once applied HD‐MAPs were left in place for 2 min immediately after which penetration was confirmed by TEWL. The subjects were then asked to come back for measurements at 24, 48 and 72 h for further histamine application. Reactions were captured by questions to the subjects regarding local sensation (itch), by naked eye observation for 10 min after histamine challenge and objectively using polarised reflectance spectroscopy.

### 2.2 TEWL

Measurements of water vapour loss from the skin were performed before and 2 min after HD‐MAP application and then again after 24, 48 and 72 h. At each measurement, the Tewameter probe was placed over the HD‐MAP application site, and measurements were collected every second for 30 s and stabilised values selected for analysis. Collected data were analysed by CK‐MPA‐Multi‐Probe‐AdapterFB, Version 2.4.2.1/207‐11‐10 (Courage ± Khazaka electronic GmbH, Köln, Germany).

### 2.3 Polarisation reflectance spectroscopy

Red blood cell concentration (RBC) was indirectly observed using Tissue Viability Imager (TiVi) (TiVi700 2.0 Tissue Viability Imager, WheelsBridge AB, Linköping, Sweden) at 2 min, 24, 48 and 72 h as well as before and after the histamine challenges to normal skin and HD‐MAP areas. The technique has previously been described in detail.^16^ Image analysis and calculation of TiVi‐values were made using WheelsBridge AB Software, version 1.2.20, November 2018, Linköping, Sweden. The tissue viability imager was set to single photo mode and medium resolution and positioned at approximately 25 cm above the observed site.

### 2.4 Histamine itch test

To determine whether the barrier function of the skin had returned, a single drop of 10 mg/ml histamine dihydrochloride (Soluprick, ALK‐Abello, Hørsholm, Denmark) was applied to an untreated area of skin as a control and directly on to the HD‐MAP application site at 24, 48 and 72 h post‐HD‐application. The drop was left on the skin for 30 s before being removed by absorption onto tissue paper, and the subsequent skin reaction was monitored. This was done to alternating arms. Thus, the 72‐h patch had been previously tested at 24 h. In the normal use of the positive histamine control test in Type I skin allergy testing, flare and wheal are accompanied by itch. In our challenge, the skin penetration manoeuvre was more severe than the standard lancet prick. Because of this, the immediate HD‐MAP application was not challenged because of an expected, and in preliminary experiments confirmed, marked reaction to HD‐MAP application. The reactions to the histamine challenge were observed for 10 min and reported as to presence of wheal clinically, presence of flare and increased central erythema in TiVi images as well as occurrence of itch. Subjects were offered antihistamines to alleviate any itch, but they were not required by any subject.

### 2.5 Ethics statement

The study ‘Minimally invasive studies of the skin's innate reactivity‐microneedle provocation’ was approved by the Linköping ethical board (MB 2017‐409‐31). The study described has been carried out in accordance with the code of ethics of the world medical association (declaration of Helsinki) for experiments involving humans.

### 2.6 Statistical analysis

TEWL and TiVi data before and after histamine challenge were compared using repeated‐measures one‐way analysis of variance (ANOVA), mixed effect analysis, *α* = 0.05 (GraphPad Prism version 9.1.2 for Windows, GraphPad Software, San Diego, California USA, www.graphpad.com).

## 3 RESULTS

### 3.1 Quantitative analysis of trans‐epidermal water loss following HD‐MAP application

**FIGURE 2:**
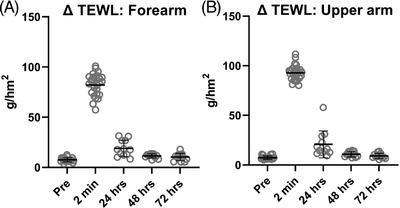
Measurement of transepidermal water loss (TEWL) from (A) the forearm and (B) upper arm following high‐density microarray patch (HD‐MAP) application (*n* = 24). Twenty‐four, 48 and 72 (*n* = 12) h measurements were done on 12 sites. The elevated values confirmed that penetration of skin barrier has occurred, and that barrier recovery ensued. Black bars represent means and standard deviation

Prior to HD‐MAP application, baseline readings of 5.7–11.1 g/hm^2^ were observed at both the upper arm and forearm application sites. Following HD‐MAP application, all sites showed an increased TEWL. The TEWL reading was at its highest in the 2‐min measurement with readings ranging between 57.3–100.9 g/hm^2^ and 81.2–111.7 g/hm^2^ for the forearm and upper arm respectively, confirming successful perturbation of the skin's barrier function. TEWL levels at 24 h were lower, although still above baseline for all sites in all patients (Figure 2). By 48‐h TEWL readings had returned to, and remained at, baseline. The same was observed at the 72‐h reading in all patients. The kinetics of water loss from the skin were thus similar to those we have previously reported.^12^

### 3.2 Kinetics of return of barrier function after histamine provocation

No urtication was seen at any time in any subject.

**FIGURE 3:**
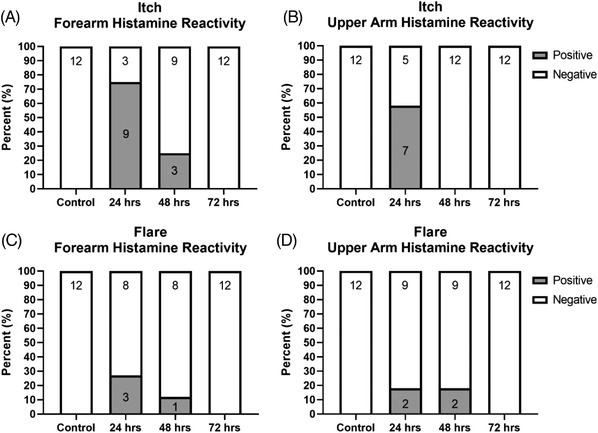
Histamine challenge at 24, 48 and 72 h failed to cause a wheal in any subject at any time point. Itch was not reported in the 72‐h provocations (A and B) but did occur in a small proportion of earlier cases. Naked eye assessment of the TiVi images (C and D) showed the occurrence of a microarray patch (MAP)‐edge flare in a small number of sites at 24 and 48 h but not at 72 h. The numbers within the bars represent number of observations

As shown in Figure 3A,B nine of 12 and seven of 12 subjects at the 24‐h time point experienced itch at the forearm and upper arm, respectively, in response to histamine provocation. However, by 48 h (when all TEWL reading had returned to baseline), only three subjects in the forearm group and one in the deltoid group reported an itch sensation. At 72 h, no subject reported itch after histamine at either site.

As seen in detail in Figure 3C,D, the TiVi images did indicate flare in a smaller proportion of volunteers at 24 h, but the proportion had decreased at 48 h and was not seen at 72 h.

**FIGURE 4:**
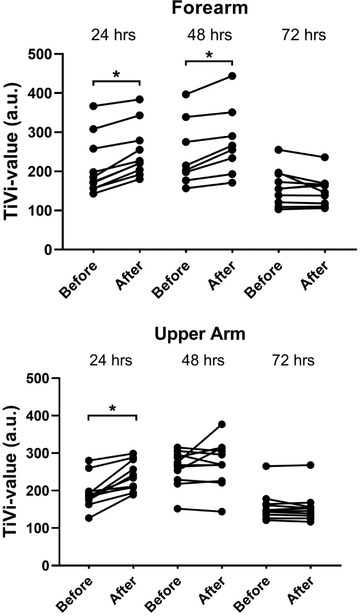
To assess the possible effects of histamine on the degree of erythema within the high‐density microarray patch (HD‐MAP) application area, TiVi data before and after histamine challenge were compared using repeated‐measures one‐way ANOVA, mixed effect analysis, *α* = 0.05. Asterisks mark significant changes

To study whether the residual erythema within the HD‐MAP application area was influenced by the histamine challenge, TiVi values before and after histamine challenge were compared in Figure 4. At 24 h, the mean TiVi within the application area at the forearm and upper arm increased in average by 13% and 14%, respectively. At 48 h, average increase was 10% and 4%, and at 72 h, there was an average slight decrease of erythema of 1% and 3% for the forearm and upper arm site, respectively.

## 4 DISCUSSION

As HD‐MAPs and other micro patch technologies make their way from the laboratory to the clinic, it becomes increasingly important to understand the physical interaction, reactivity and recovery of the skin following patch application. We have performed a study investigating the skin's recovery to HD‐MAP‐induced microtrauma at two sites, volar forearm and deltoid region in terms of return towards the resistance to topical histamine challenge of normal unprovoked skin. The 2 min post‐application TEWL values confirmed an adequate provocation. A minority of the 48 h reactions itched, demonstrating an individual variability which we have also seen in other aspects of MAP reactivity.^12^ A majority of the 24 h applications to the forearm gave itch with slightly fewer of the deltoid applications itching.

In our previous study of HD‐MAP provocation in normal skin, we had concluded that the skin abrogation had returned to normal by 48 h, the chief evidence being return of TEWL to near normal levels.^12^ The present study reports the outcome of the topical application of histamine to the provocation area, a challenge demonstrated to produce no itching, erythema or urtication in normal skin. TEWL is an indirect measurement compared to the direct histamine provocation. There is no universal definition for what constitutes an intact skin barrier. We know from the study of other minimal trauma models that in early healing the barrier can constitute one layer of keratinocytes.^17^ How thick or well differentiated a keratinocyte layer needs to be to reach full barrier function is unclear. Although histamine provocation may be a more sensitive test for the demonstration of a completely intact barrier, it can be associated with discomfort, and we feel that evaporimetry is the preferred method for following skin barrier recovery (healing). Histamine being a small molecule is likely to be a ‘worst case’ example of barrier penetration. The present paper shows that after due consideration of individual subject variability, the two methods are in general agreement and support our original contention of micropuncture closure by 48 h.

The strength of an individual's innate immune reactivity influences the degree of response to minimal trauma such as HD‐MAP application. The innate immune response also has an important role in the complex process of wound healing in which barrier restoration, the focus of the present paper, is achieved.^18^, ^19^, ^20^ We plan further studies to elucidate reactivity and healing in subgroups of varying age which can be suspected of having decreased reactivity and healing capability.

The paper further confirms the broad occurrence of reactivity, which is considered necessary for effective vaccination, and the rapid restoration of barrier function against even a small molecule like histamine demonstrates that although this study was conducted on healthy individuals, the vaccination site, at which a maximal area of 1 cm^2^ represents skin perturbation, should not be at extended risk of infection even in individuals with any background disease such as diabetes.

## 5 CONCLUSION

Our findings support the contention that micropuncture closure after HD‐MAP application occurs in most subjects by 48 h.

## CONFLICT OF INTEREST

S. Ben Baker is a Vaxxas employee. David A. Muller and Chris D Anderson consult for Vaxxas. Joakim Henricson has a royalty agreement with and Chris D. Anderson is a share holder of the company WheelsBridge AB making the TiVi equipment. Remaining authors declare no conflict of interest.
